# Calreticulin promotes EMT in pancreatic cancer via mediating Ca^2+^ dependent acute and chronic endoplasmic reticulum stress

**DOI:** 10.1186/s13046-020-01702-y

**Published:** 2020-10-07

**Authors:** Weiwei Sheng, Guosen Wang, Jingtong Tang, Xiaoyang Shi, Rongxian Cao, Jian Sun, Yi Heng Lin, Chao Jia, Chuanping Chen, Jianping Zhou, Ming Dong

**Affiliations:** 1 Department of Gastrointestinal Surgery, The First Hospital, China Medical University, Shenyang, 110001 Liaoning China; 2 Department of General Surgery, The First Affliated Hospital, Nanchang University, Nanchang, 330006 Jiangxi China; 3 Department of General Surgery, The People’s Hospital of Liaoning province, Shenyang, 110034 Liaoning China; 4 Department of Clinical Laboratory, The Sixth Peoples’ hospital of Shenyang, Shenyang, 110003 Liaoning China

**Keywords:** Calreticulin, Intracellular free Ca^2 +^, Endoplasmic reticulum stress, IRE1α, Epithelial mesenchymal transition, Pancreatic cancer

## Abstract

**Background:**

Our previous study showed that calreticulin (CRT) promoted EGF-induced epithelial-mesenchymal transition (EMT) in pancreatic cancer (PC) via Integrin/EGFR-ERK/MAPK signaling. We next investigated the novel signal pathway and molecular mechanism involving the oncogenic role of CRT in PC.

**Methods:**

We investigated the potential role and mechanism of CRT in regulating intracellular free Ca^2+^ dependent acute and chronic endoplasmic reticulum stress (ERS)-induced EMT in PC in vitro and vivo.

**Results:**

Thapsigargin (TG) induced acute ERS via increasing intracellular free Ca^2+^ in PC cells, which was reversed by CRT silencing. Additionally, CRT silencing inhibited TG-induced EMT in vitro by reversing TG-induced changes of the key proteins in EMT signaling (ZO-1, E-cadherin and Slug) and ERK/MAPK signaling (pERK). TG-promoted cell invasion and migration was also rescued by CRT silencing but enhanced by IRE1α silencing (one of the key stressors in unfolded protein response). Meanwhile, CRT was co-immunoprecipitated and co-localized with IRE1α in vitro and its silencing led to the chronic ERS via upregulating IRE1α independent of IRE1-XBP1 axis. Moreover, CRT silencing inhibited IRE1α silencing-promoted EMT, including inhibiting the activation of EMT and ERK/MAPK signaling and the promotion of cell mobility. In vivo, CRT silencing decreased subcutaneous tumor size and distant liver metastasis following with the increase of IRE1α expression. A negative relationship between CRT and IRE1α was also observed in clinical PC samples, which coordinately promoted the advanced clinical stages and poor prognosis of PC patients.

**Conclusions:**

CRT promotes EMT in PC via mediating intracellular free Ca^2+^ dependent TG-induced acute ERS and IRE1α-mediated chronic ERS via Slug and ERK/MAPK signaling.

## Background

Pancreatic cancer (PC) is one of the most aggressive and lethal cancers, with an estimated 55,440 new cases and 44,330 deaths in United States in 2018 [1]. The strong ability of local invasion and rapid metastasize are major hallmarks of PC, which contribute to the poor prognosis of patients. Thus, it is urgent to reveal the molecular mechanisms and target therapies toward the malignant biology and aggressive progression in PC.

Calreticulin (CRT), as a highly conserved endoplasmic reticulum (ER) Ca^2+^-buffering chaperone, involves in various cellular processes [2]. We previously reported that CRT overexpression promoted cell invasion, migration and drug resistance of PC by activating ERK/MAPK pathway [3]. Most recently, we showed that CRT silencing inhibited EGF-induced epithelial-mesenchymal transition (EMT) via the Integrin/EGFR-ERK/MAPK pathway in PC [4]. Based on previous studies, we next investigated the novel signaling pathways and molecular mechanisms involving the oncogenic role of CRT in PC development.

Endoplasmic reticulum stress (ERS) is a defensive response induced by various pathophysiological factors, which is triggered by three transmembrane signal transducers from unfolded protein response (UPR) family: PKR-like endoplasmic reticulum kinase (PERK), inositol-requiring enzyme 1α (IRE1α) and activating transcription factor-6 (ATF-6) [5]. ERS plays a significant role in tumor biology including EMT mediated tumor invasion and metastasis [6]. However, the definite role of ERS in malignancies remains controversial [7]. Emerging evidences suggest that ERS plays a dual role in tumor progression. A transient ERS response activates a protective function and pro-survival pathway to cancers, whereas long term ERS triggers death signaling [8, 9].

Intracellular free Ca^2+^ is a multifunctional second messenger that controls diverse cellular functions [10]. Recently, we have reported that alteration of CRT mediates intracellular free Ca^2+^ concentration in PC cells [4]. Moreover, dysfunction of cellular Ca^2+^ homeostasis is a main stimulator of ERS [11], which is closely related with cell invasion, immune evasion, EMT and drug resistance in various cancers [12]. Therefore, we sought to evaluate the potential role of CRT in Ca^2+^ homeostasis mediated ERS and EMT in PC, which, to our knowledge, has not been reported yet.

## Materials and methods

### Tissue samples and cell lines

This study was approved by the academic committee at the First Hospital of China Medical University. Written informed consent has been obtained from each patient. Eight-one pancreatic ductal adenocarcinoma (PDAC) tissues were procured from surgical resection specimens collected by the Department of Gastrointestinal Surgery at the First Hospital, China Medical University.

Human Capan-2 PC cell line was obtained from the American Type Culture Collection (ATCC, Manassas, VA, USA). SW1990 human PC cell line was purchased from the Cell Bank of the Chinese Academy of Sciences (Shanghai, China). Cells were cultured with recommended growth media with 10% fetal bovine serum (FBS, HyClone, Logan, UT, USA).

### Fluo-3 assay

Thapsigargin (TG, Sigma, St Louis, MO, USA) is one of the key stimulators that cause acute ERS via specific inhibiting sarcoplasmic/endoplasmic reticulum Ca^2+^-ATPases (SERCAs), resulting in an increase of cytoplasmic Ca^2+^ concentration [13]. The intracellular free Ca^2+^ concentration was measured using Fluo-3 AM (Beyotime, Shanghai, China), according to the manufacturer’s instructions. Briefly, transfected PC cells were pretreated with 200 nM TG and 1% DMSO (control) for 4 h. Cells without or with TG treatment were subsequently loaded with 2 μM Fluo-3 AM for 30 min at 37 °C and then washed with Hanks’ Balanced Salt Solution (HBSS, Beyotime) for 3 times. Kept incubating with HBSS for 20 min, the fluorescence was visualized on a confocal microscopy (Leica Tcs Sp5 II, Leica, Heidelberg, Germany) at an excitation wavelength of 488 nm with an emission wavelength of 525 nm.

In addition, cells without or with TG stimuli were harvested by pancreatic enzymes without EDTA, washed by HBSS for 3 times, and then submitted to analysis by flow cytometry. Image analysis was performed using the Image J software. Each experiment was repeated 3 times.

### Immunohistochemistry (IHC) assay

As described previously [4, 14], 4-μm sections were covered with 0.3% H_2_O_2_, subjected to high pressure, added with goat serum, and then incubated with primary antibodies: CRT (Abcam, Cambridge, UK) and IRE1α (Cell Signaling Technology, CST, Beverly, MA, USA). Then the slices were incubated with the secondary antibodies, treated with streptavidin–peroxidase reagent, visualized with DAB, counterstained with hematoxylin and finally evaluated under microscope. The location of CRT and IRE1α in cytoplasm were considered for scoring. Staining intensity was scored as 0–3 (negative, weak, medium and strong). Extent of staining was scored as 0 (< 5%), 1 (5–25%), 2 (26–50%), 3 (51–75%), and 4 (> 75%) according to the positive staining areas to the whole carcinoma. The final scores were calculated by 3 pathologists. We used the same scoring method to evaluate the IHC assay in vivo and in human PDAC specimens.

### Immunofluorescence (IF) staining

Capan-2 and SW1990 cell lines were implanted into 24-well culture plates covered with slices, fixed in 4% paraformal dehyde, permeabilized with Triton X-100 (0.5%) and incubated with 5% BSA. Then plates were incubated with the primary antibodies overnight: CRT (Abcam) and IRE1α (Cell signaling technology). The secondary antibodies (Proteintech, Chicago, IL, USA) were conjugated with FITC for CRT and TRITC for IRE1α. Hoechest33258 were used for nuclear visualizing.

### Western blot (WB) assay

Whole protein lysates were prepared from transfected PC cells. Samples were loaded onto 10% SDS-polyacrylamide gels, transferred to PVDF membranes and incubated with primary antibodies: CRT (Abcam), IRE1α (CST), PREK (CST), phosphorylation PKR-like endoplasmic reticulum kinase (p-PERK, CST), ATF-6 (CST), ZO-1 (Proteintech), ZEB1 (Proteintech), N-cadherin (Proteintech), E-cadherin (Proteintech), Vimentin (Proteintech), phosphorylation extracellular regulated protein kinases (pERK, CST), extracellular regulated protein kinases (ERK, CST), X-box-binding protein 1 (XBP1, Proteintech), Snai1 (Proteintech), Slug (CST), Cavelino-1 (Proteintech), GAPDH (Proteintech) and β-actin (Proteintech) antibodies overnight at 4 °C. Then, membranes were incubated with secondary antibodies (Santa Cruz, CA, UK) and finally detected with an ECL detection kit (Thermo Scientific, Rockford, IL, USA). The experiments were repeated for 3 times.

### Coimmunoprecipitation (CoIP) assay

CoIP was performed as before [4, 14]. Briefly, PC cells were lysed in lysis buffer and the soluble supernatants were isolated. Magnetic beads (Bio-Rad, California, USA) were preincubated with primary CRT (Abcam), IRE1α (CST) or IgG (Santa Cruz) antibodies at 4 °C for 4 h with rotation. Then antibody-beads complexes were incubated with soluble supernatants at 4 °C overnight. Immunoprecipitated proteins were analyzed by WB with a variety of antibody.

### CRISPR/Cas9 and siRNA mediated silencing of CRT and IRE1α

Lentiviruses were synthesized by Genechem (Shanghai, China). PC cells were transfected with lenti-cas9 or lenti-sgRNA as described previously [4, 14], and then screened using puromycin (Sigma). The stable sub-lines were subsequently transfected with sg1-CRT or sg2-CRT to specifically silence the target gene or an sgRNA control (scramble).

IRE1α siRNA and siRNA control were synthesized from GenePharma (Shanghai, China). Cells were transiently transfected with siRNA (20 μM) using oligofectamine3000 (Invitrogen, Carlsbad, CA, USA) as described by the protocol. All target sequences mentioned above were shown in Supplemental Material Table 1.

### TG induced EMT construction

Stable transfected PC cells were treated with 200 nM TG or 1% DMSO (as a control) for 4 h. The EMT construction was verified by EMT-enhanced cell invasion and migration and EMT-induced changes in key proteins involving in EMT signaling.

### Invasion and migration assays

Briefly, transfected PC cells (pretreated with TG or co-transfected with IRE1α) were plated in inserts that coated with matrigel (BD Biosciences, Sparks, MD, USA) in 24 well plates with FBS-free growth media. Growth media with 10% FBS was added to the bottom wells to generate a serum gradient. After 24 h, cells that had migrated to the underside of the inserts were stained with Crystal Violet Hydrate (Sigma). The migratory cells were counted in five random fields per well. The migration assay was done in a similar fashion without matrigel. Each experiment was repeated 3 times.

### In vivo xenograft model

All animal work was performed in accordance with protocols approved by the Animal Care Committee of China Medical University. Total 15 nude mice (BALB/c-nu) were used. Transfected Capan-2 cells (1 × 10 [6]) were respectively injected into bilateral axillae of 5 nude mice to construct subcutaneous tumor formation. Tumor volumes were calculated by the following formula: length × width × height × 0.52 in cm. Besides, transfected SW1990 cells (1 × 10 [6]) were injected into the spleen of 10 nude mice to construct distant liver metastasis model, which were assessed by the number of liver metastases. These nude mice were killed 30 days later, and samples were extracted and fixed for hematoxylin and eosin (HE), and IHC staining.

### Statistical analysis

Statistical analysis was performed using SPSS software 21.0 (Chicago, IL, USA). Continuous variables were expressed as the mean ± SD. The differences in intracellular free Ca^2+^ concentration, WB assay, cell migration and invasion assays and the number of liver metastases were compared through Student’s t-test. The differences of orthotopic tumor volumes were compared with paired sample t-test. Non-parametric and spearman correlation tests were analyzed for IHC assays in vivo and human PC samples. The association of target proteins expression with clinicopathological data was analyzed by Chi-squared. The Kaplan-Meier curve was used to estimate survival, and differences were analyzed by the log-rank test. *P* < 0.05 or *P* < 0.01 was considered significant.

## Results

### CRT silencing inhibited TG-induced increase of intracellular free Ca^2+^ concentration in vitro

**Fig. 1:**
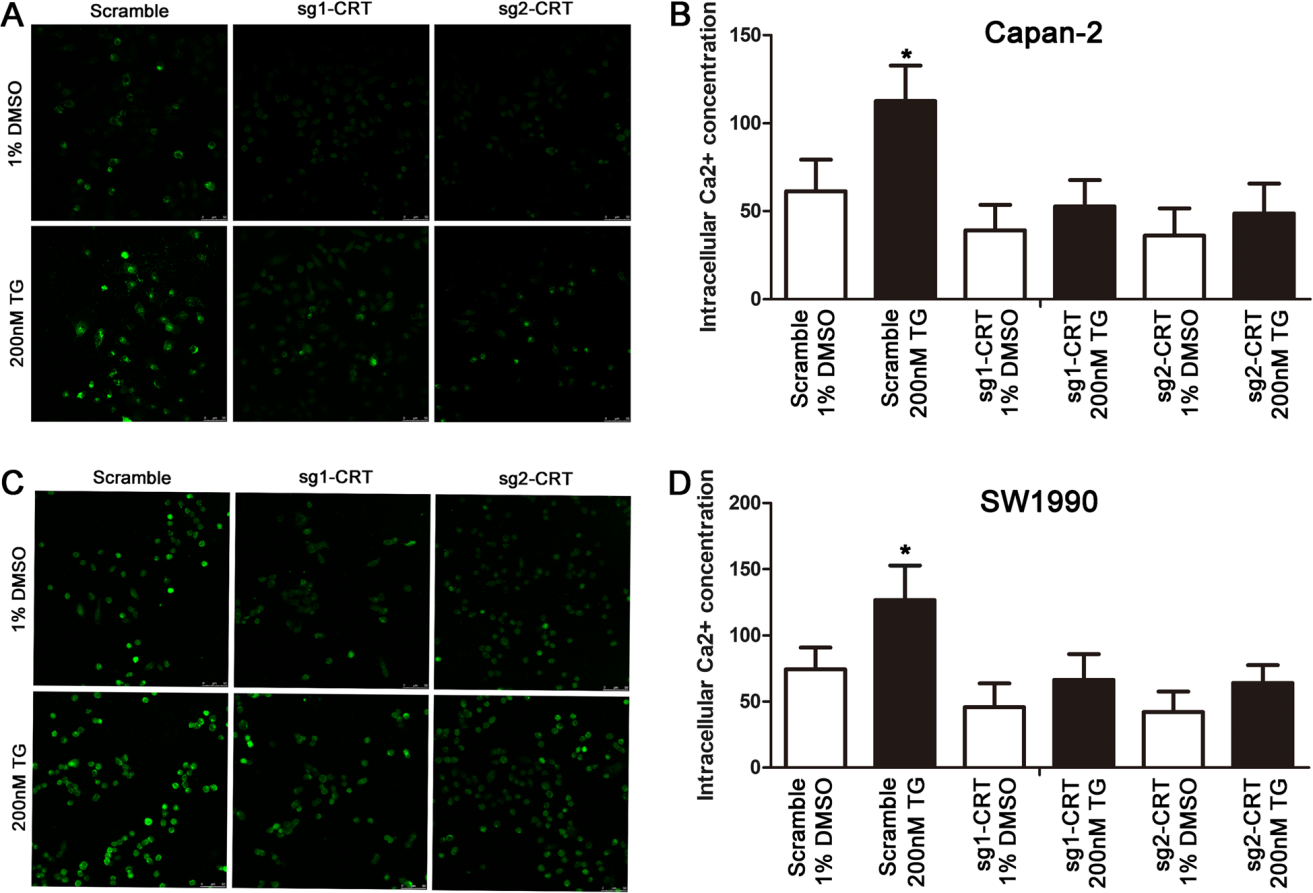
CRT silencing inhibited TG-induced the increase of intracellular free Ca^2+^ concentration in vitro. **a, b** The intracellular free Ca^2+^ concentration in Capan-2 cells detected by confocal microscopy (**a**) or flow cytometry (**b**), respectively. **c, d** The intracellular free Ca^2+^ concentration in SW1990 cells detected by confocal microscopy (**c**) or flow cytometry (**d**), respectively. TG: Thapsigargin. Data are shown as mean ± SD. *P < 0.05 versus control

Our previous study showed that CRT regulated intracellular free Ca^2+^ in PC cells [4]. Continuing to use the Fluo-3 assay, confocal microscopy showed that TG induced the increase of intracellular free Ca^2+^ in Capan-2 and SW1990 cells, which was significantly inhibited by CRT silencing (Fig. 1a and c). Without any stimulus, CRT silencing partially decreased intracellular free Ca^2+^ in PC cells. Upon TG, the growing gap of intracellular free Ca^2+^ between scramble groups with and without TG was much more obvious than that in sg1-CRT and sg2-CRT groups (Fig. 1a and c). Similarly, in flow cytometry assays, upon TG, the growing gap of intracellular free Ca2+ between scramble groups with and without TG was much more obvious than that in sg1-CRT and sg2-CRT groups (Fig. 1b and d). It indicates that the increase of intracellular Ca^2+^ induced by TG is partially regulated by CRT.

### CRT silencing inhibited TG-induced EMT in vitro

Intracellular free Ca^2+^, acts as the second messenger, is essential for multiple cellular functions including invasion and migration [10]. Our previous study reasoned that CRT regulated intracellular free Ca^2+^ in PC via Integrin/EGFR-ERK/MAPK, which played an important role in EGF-induced EMT. Ca^2+^ signaling-mediated EMT has also been reported in various cancers [12, 15]. However, the relationship between Ca^2+^ mediated ERS and EMT remains controversial [16, 17].

**Fig. 2:**
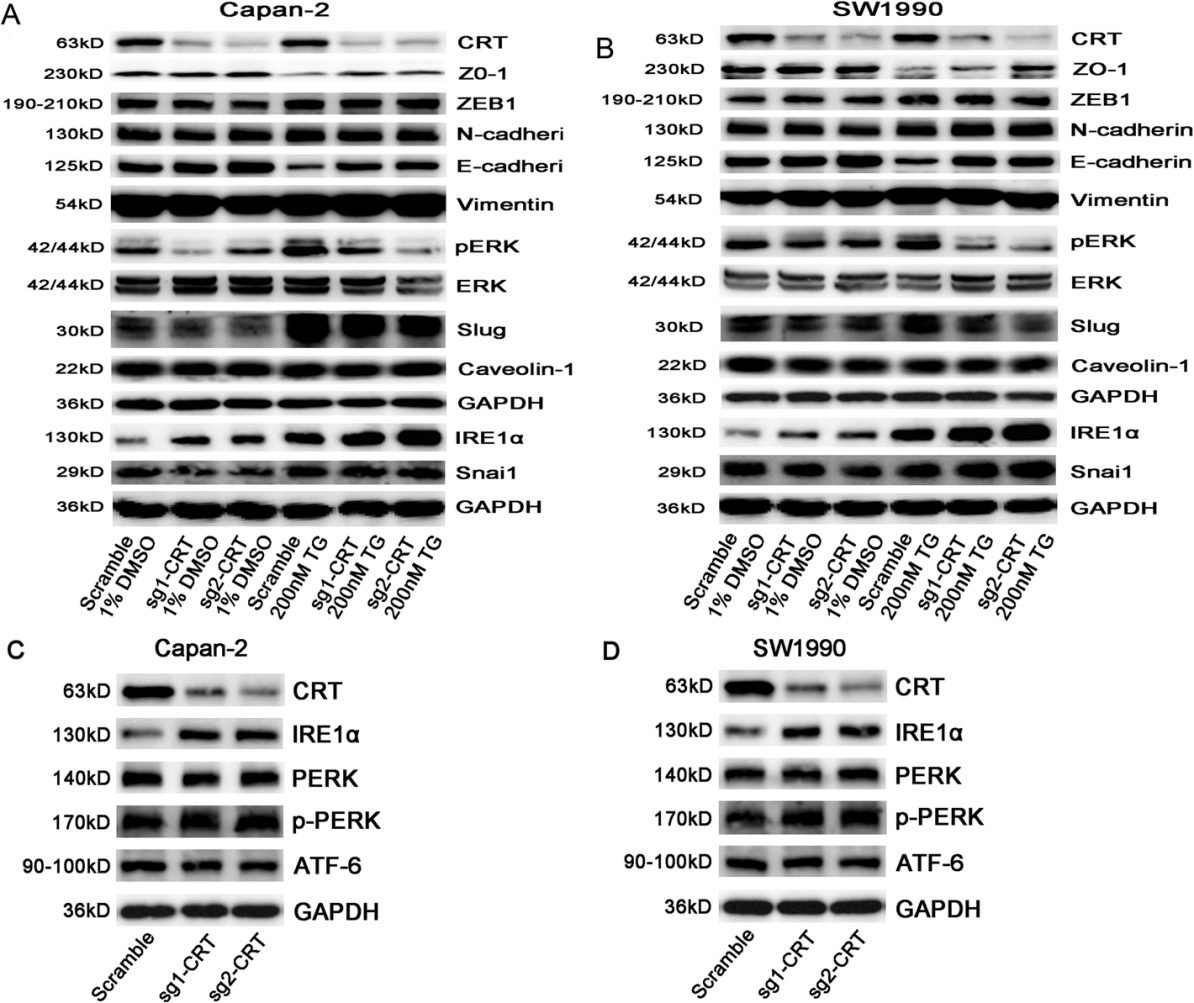
The effect of CRT silencing in TG-induced EMT and UPR activation in vitro by WB. **a** The expression of EMT classic markers in scramble, sg1-CRT and sg2-CRT transfected Capan-2 cells with or without TG treatment. **b** The expression of EMT classic markers in scramble, sg1-CRT and sg2-CRT transfected SW1990 cells with or without TG treatment. c**, d** The expression of UPR family: IRE1α, PERK and ATF-6 in CRT silencing Capan-2 (**c**) and SW1990 (**d**) cells. TG: Thapsigargin. The statistic data of WB was shown in Supplemental Fig. 1

TG treatment caused cell apoptosis in various cancers [18]. To our surprise, we found an oncogenic role of TG in PC cells via activating acute ERS. Firstly, TG activated EMT and ERK/MAPK signaling by inducing the decrease of EMT epithelial markers E-cadherin and ZO-1, and the increase of pERK and EMT key regulators Snai1, Slug and ZEB1 in Capan-2 and SW1990 cells (Fig. 2a, b and Supplemental Fig. 1). However, CRT silencing reversed TG-induced the changes of above proteins (except for ZEB1 and Snai1). In detail, without TG stimuli, E-cadherin and ZO-1 expression were slightly increased and pERK and Slug expression was partially decreased in sg-CRT group compared with scramble group. Other EMT markers, such as Snai1, ZEB-1, N-cadherin, Vimentin and Caveolin-1 were unchanged (Fig. 2a, b and Supplemental Fig. 1). However, upon TG stimuli, a significant increase of E-cadherin and ZO-1 and decrease of pERK and Slug were found in sg-CRT group compared with scramble group in both Capan-2 and SW1990 cells (Fig. 2). In addition, TG induced IRE1a expression in vitro which was one of the key stressors in UPR. Meanwhile, CRT silencing stably upregulated IRE1α expression whatever with or without TG treatment (Fig. 2a, b and Supplemental Fig. 1).

**Fig. 3:**
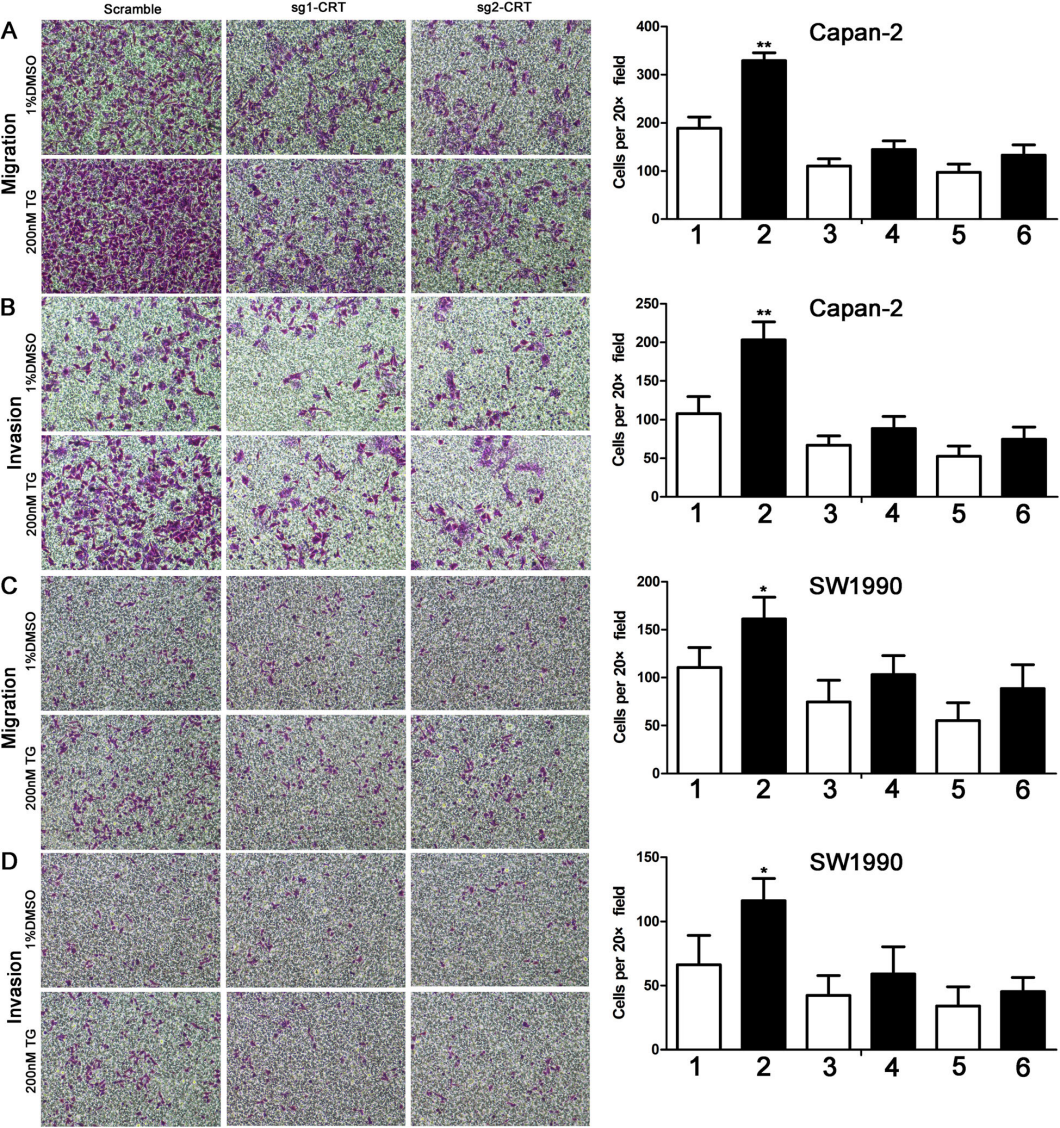
CRT silencing inhibited TG-induced the increase of cell migration and invasion in vitro. **a, b** CRT silencing inhibited TG-induced the increase of cell migration (**a**) and invasion (**b**) in Capan-2 cells. **c, d** CRT silencing inhibited TG-induced the increase of cell migration (**c**) and invasion (**d**) in SW1990 cells. TG: Thapsigargin. Data are shown as mean ± SD. *P < 0.05, **P < 0.01 versus control. 1:Scrabmle+1%DMSO;2:Scramble+200nM TG;3:sg1-CRT+1%DMSO; 4:sg1-CRT+200nM TG; 5:sg2-CRT+1%DMSO; 6:sg2-CRT+200nM TG

For cell mobility assays, TG stimulated cell invasion and migration in both Capan-2 and SW1990 cells (Fig. 3). Without TG, CRT silencing alone partially inhibited cell invasion and migration in both two cell lines. However, upon TG, a significant increase of cell invasion and migration were found in scramble groups. Namely, the growing gap of cell motility between scramble groups with and without TG was much more obvious than that in sg1-CRT and sg2-CRT groups (Fig. 3). Interestingly, IRE1α silencing enhanced TG-induced cell invasion and migration in both Capan-2 and SW1990 cells in Supplemental Fig. 2. We reasoned that the compensatory increase of IRE1α induced by TG partially inhibited TG-induced cell mobility in vitro.

Taken together, CRT silencing inhibited TG-induced acute ERS and EMT in vitro via Slug and ERK/MAPK signaling.

### CRT silencing upregulated IRE1α (one of the UPR key stressors) independent of XBP1 in vitro

**Fig. 4:**
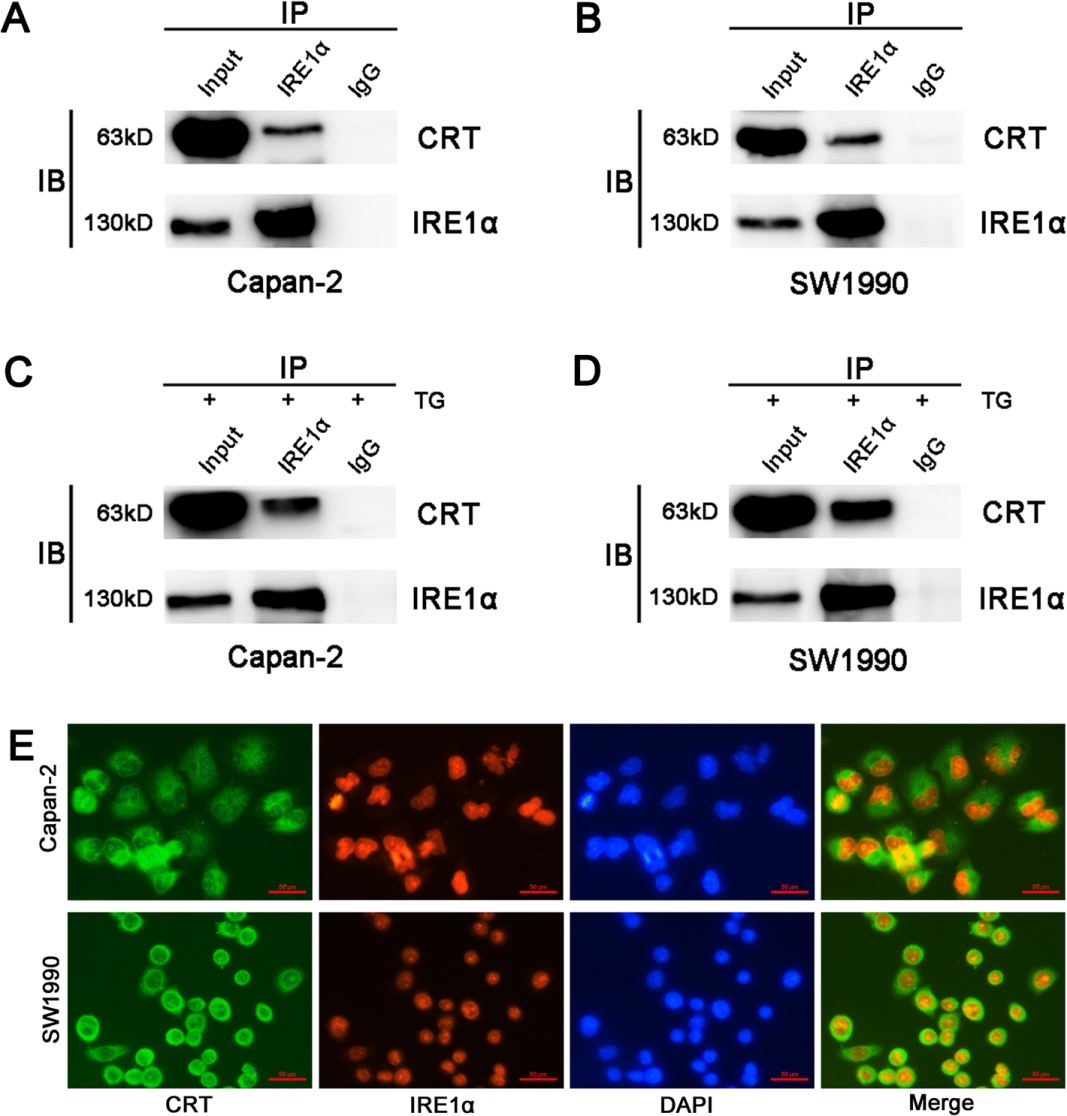
The close interaction between CRT and IRE1α in vitro detected by IP and IF. **a, c** CRT was coimmunoprecipitated with IRE1α in the lysates of Capan-2 cells without (**a**) or with (**c**) TG treatment. **b, d** CRT was coimmunoprecipitated with IRE1α in the lysates of SW1990 cells without (**b**) or with (**d**) TG treatment. **e** CRT was partially co-localized with IRE1α in Capan-2 and SW1990 cells. TG: Thapsigargin. CRT stained with FITC. IRE1α stained with TRIC

Chronic ERS produces endogenous or exogenous damage to cells and triggers an evolutionarily conserved response, termed UPR that including three major stressors located on the ER membrane: PERK, IRE1α and ATF6 [19]. As mentioned above, CRT silencing led to a stable low level of intracellular Ca^2+^ in PC cells, which contributes to chronic ERS in many cells and tissues [20, 21]. Meanwhile, CRT silencing stably upregulated IRE1α expression whatever with or without TG treatment. Thus, we next investigated the role of CRT in mediating chronic ERS and EMT in PC. WB showed that CRT silencing upregulated IRE1α expression in both Capan-2 and SW1990 cells, but had no effect in PERK and ATF6 expression (Fig. 2c, d and Supplemental Fig. 1). Meanwhile, CRT was co-immunoprecipitated with IRE1α in the lysates of above cell lines whatever with or without TG treatment (Fig. 4a-d). IF further showed the partial co-location of CRT and IRE1α in vitro (Fig. 4e). Thus, a specific interaction between CRT and IRE1α was observed in CRT-mediated chronic ERS.

**Fig. 5:**
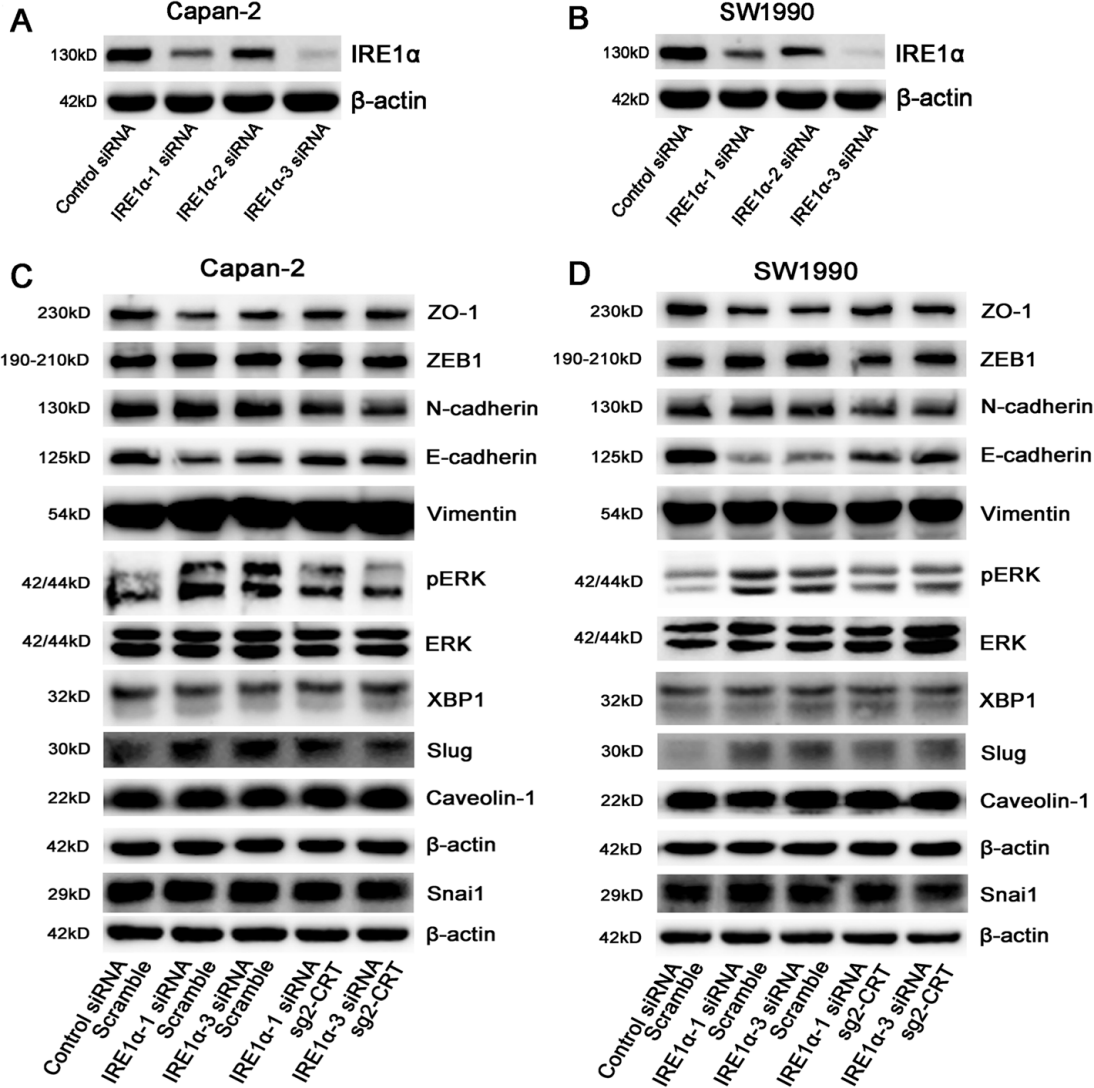
CRT silencing inhibited IRE1α silencing-induced the changes of EMT markers in vitro. **a, b** The expression of IRE1α in control siRNA, IRE1α-1 siRNA, IRE1α-2 siRNA and IRE1α-3 siRNA transfected Capan-2 (**a**) and SW1990 (**b**) cells. **c, d** The expression of EMT classic markers in Capan-2 (**c**) and SW1990 (**d**) cells transfected with the combinations shown. The statistic data of WB was shown in Supplemental Fig. 3

**Fig. 6:**
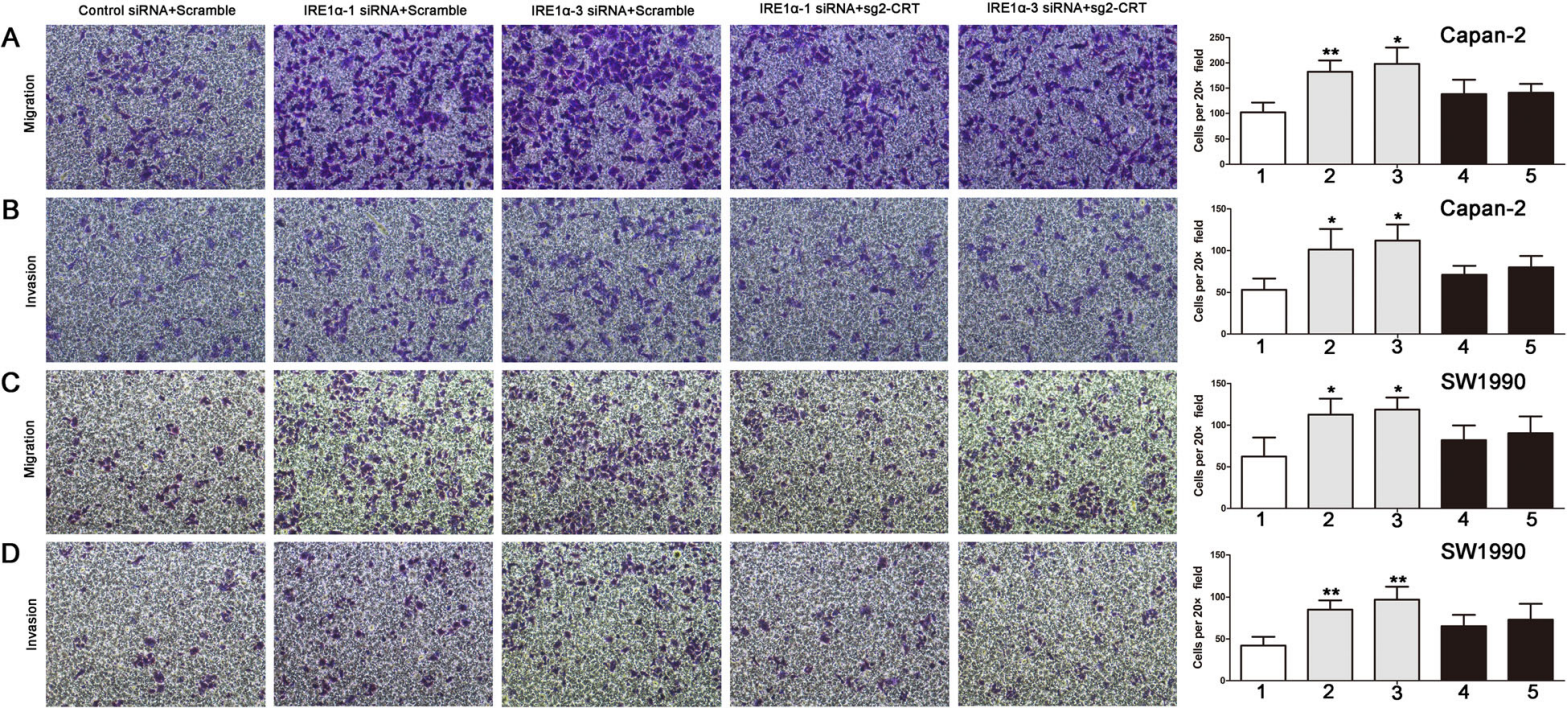
CRT silencing inhibited IRE1α silencing-induced the increase of cell migration and invasion in vitro. **a, b** Cell migration (**a**) and invasion (**b**) in Capan-2 cells transfected with the combinations shown. **c, d** Cell migration (**c**) and invasion (**d**) in SW1990 cells transfected with the combinations shown. Data are shown as mean ± SD. *P < 0.05, **P < 0.01 versus control. 1: Control siRNA+Scramble; 2:  IRE1α-1 siRNA+Scramble; 3: IRE1α-3 siRNA+Scramble: 4:  IRE1α-1 siRNA+sg2-CRT; 5: IRE1α-3 siRNA+sg2-CRT

IRE1α acts as an oncogene in prostate cancer and glioblastoma [22, 23]. It is responsible for alternative splicing of the XBP1 transcription which induces Snail expression to promote EMT in breast cancer cells [24]. Therefore, we next investigated whether CRT mediated chronic ERS-induced EMT via regulating IRE1/XBP1 axis. We first found that IRE1α protein was much lower in IRE1α siRNA group compared with control siRNA group, especially in IRE1α-1 siRNA and IRE1α-3 siRNA groups (Fig. 5a, b and Supplemental Fig. 3). To our surprise, IRE1α silencing promoted EMT in PC cells via Slug and ERK/MAPK signaling. In Capan-2 and SW1990 cells, IRE1α silencing promoted Slug and pERK expression and inhibited E-cadherin and ZO-1 expression, but had no effect in Snai1, ZEB-1, N-cadherin, Vimentin and Caveolin-1 expression (Fig. 5c, d). XBP1, as a spliced target by IRE1α, was also unchanged. However, CRT silencing reversed IRE1α siRNA mediated changes in EMT and ERK/MAPK signaling independent of IRE1α/XBP1 axis (Fig. 5c, d). Downregulation of E-cadherin and ZO-1 and upregulation of Slug and pERK were less significant in IRE1α siRNA plus sg2-CRT group, compared with that in control siRNA plus scramble group (Fig. 5c, d). In addition, IRE1α silencing significantly promoted cell invasion and migration in PC cells, which was also significantly reversed by CRT silencing (Fig. 6). In detail, compared with control siRNA plus scramble group, the growing gap of cell motility in IRE1α siRNA plus scramble group was much more significant than that in IRE1α siRNA plus sg2-CRT group (Fig. 6). Taken together, CRT silencing inhibited IRE1α silencing-induced chronic ERS and EMT in vitro via Slug and ERK/MAPK signaling independent of IRE1α/XBP1 axis.

### CRT silencing inhibited subcutaneous tumor size and distant liver metastasis in vivo

**Fig. 7:**
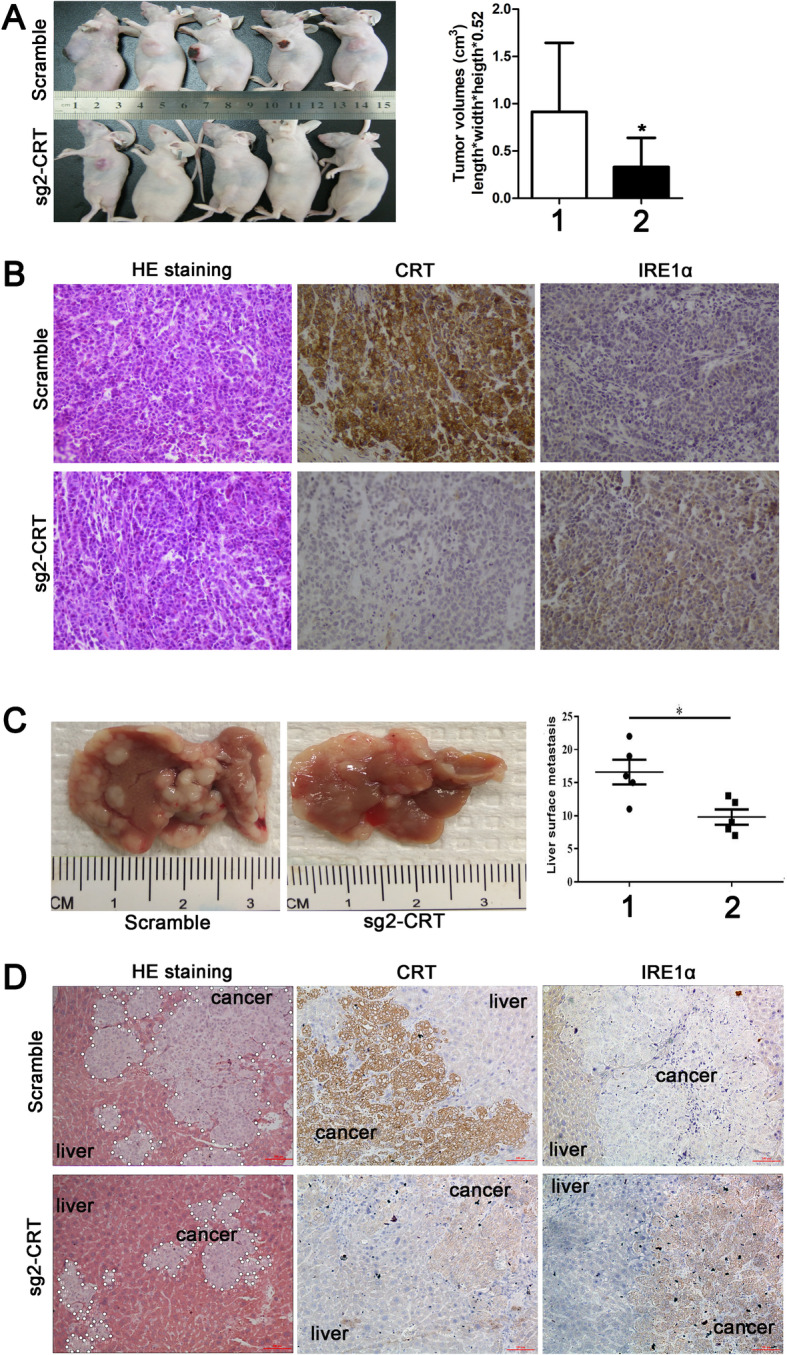
CRT silencing inhibited subcutaneous tumor size and distant liver metastasis in vivo. **a** The representative images and statistical data of tumor volumes between scramble and sg2-CRT groups in nude mice. **b** The representative HE and IHC images of CRT and IRE1α expression in subcutaneous tumor between scramble and sg2-CRT groups. **c** The representative images and statistical data of the number of liver metastases between scramble and sg2-CRT groups in nude mice. **d** The representative HE and IHC images of CRT and IRE1α expression in distant liver metastasis between scramble and sg2-CRT groups. Data are shown as mean ± SD. *P < 0.05 versus control. Data are shown as mean ± SD. *P < 0.05 versus control.1: Scramle; 2: sg2-CRT

Capan-2 cells (derived from primary PC) were used to construct subcutaneous tumorigenesis model in bilateral axillae of the nude mice. Tumor volumes in sg2-CRT group were much smaller than that in paired scramble group (Fig. 7a). HE staining confirmed the tumor pathology in both groups (Fig. 7b). IHC further verified that CRT expression was significantly decreased but IRE1α was increased in sg2-CRT group compared with the scramble group, and vice versa (Fig. 7b).

SW1990 cells (derived from spleen metastasis) were used to construct liver metastasis model in nude mice. The number of liver metastases in sg2-CRT group were less than that in scramble group (Fig. 7c). HE staining also showed a large and serial area of liver metastasis in scramble group compared with that in sg2-CRT group (Fig. 7d). IHC further verified that CRT expression was significantly deceased but IRE1α was increased in sg2-CRT group compared with the scramble group, and vice versa (Fig. 7d).

### A negative expression between CRT and IRE1α cooperatively affected the survival of PC patients

**Fig. 8:**
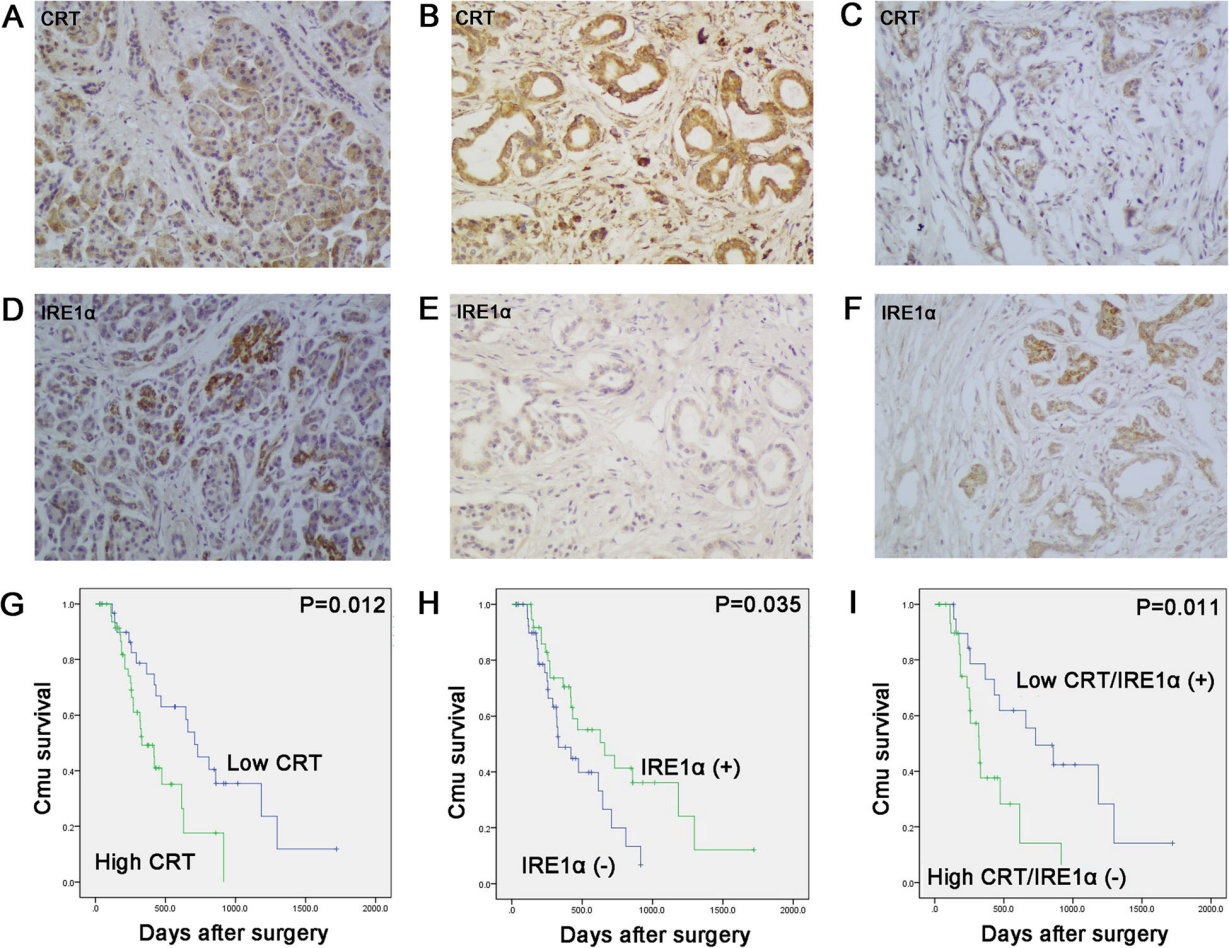
The negative relationship of CRT and IRE1α expression in human PC samples was coordinately associated with the survival of PC patients. **a, d** Low CRT (**a**) and positive IRE1α (**d**) expression in normal pancreas. **b, e** High CRT (**b**) and negative IRE1α (**e**) expression in one serial PC sample. **c, f** Low CRT (**c**) and positive IRE1α (**f**) expression in another serial PC sample. **g** Kaplan–Meier curves for patients with high versus low CRT expression in tumors. **h** Kaplan–Meier curves for patients with positive (+) versus negative (−) IRE1α expression in tumors. **I** Kaplan–Meier curves for patients with the co-expression of CRT and IRE1α

Finally, we investigated the close relationship between CRT and IRE1α with the clinicopathological data of PC patients. CRT was overexpressed (50/81; 61.7%), but IRE1α was downregulated (38/81; 46.9%) in 81 PC samples (Fig. 8a-f). CRT overexpression was positively associated with lymph nodes metastasis and UICC stage (*P* = 0.003 and *P* = 0.004, respectively), while IRE1α positive expression was negatively associated with lymph nodes metastasis and UICC stage (*P* = 0.012 and *P* = 0.002, respectively) (Table 1). A negative expression between CRT and IRE1α was also observed in human PC samples (r = − 0.278; *P =* 0.012) (Table 2). In serial sections, most PC tissues with high CRT expression trended to be associated with negative IRE1α expression (Fig. 8b, e), and vice versa (Fig. 8c, f).

**Table 1:** Association of CRT and IRE1α expression with clinicopathological data in PC patients

Parameters	No. of patients	CRT		P	IRE1α		P
		Low	High		Negative	Positive	
Cases	81	31	50		43	38	
Age (years)							
≤ 65	63	25	38	0.625	36	27	0.171
> 65	18	6	12		7	11	
Gender							
Male	47	19	28	0.639	21	26	0.075
Female	34	12	22		22	12	
Tumor location							
Head	57	21	36	0.683	30	27	0.899
Body-tail	24	10	14		13	11	
Tumor size (cm)							
< 3	24	7	17	0.274	13	11	0.899
≥ 3	57	24	33		30	27	
Differentiation							
Well	22	7	15	0.466	12	10	0.872
Moderate and poor	59	24	35		31	28	
T stage							
T1 + T2	72	30	42	0.157	37	35	0.609
T3 + T4	9	1	8		6	3	
Lymph nodes metastasis							
N0 (negative)	65	30	35	0.003	30	35	0.012
N1 (positive)	16	1	15		13	3	
UICC stage							
I + IIA	62	29	33	0.004	27	35	0.002
IIB + III	19	2	17		16	3	
Perineural invasion							
Absent	70	28	42	0.636	37	33	0.917
Present	11	3	8		6	5	
Vascular permeation							
Absent	49	15	34	0.079	28	21	0.365
Present	32	16	16		15	17	
Pre-therapeutic CA19–9 level (U/ml)							
< 37	22	12	10	0.066	8	14	0.066
≥ 37	59	19	40		35	24	
Postoperative liver metastasis							
Negative	54	23	31	0.258	27	27	0.431
Positive	27	8	19		16	11

**Table 2:** Correlation analysis of the relationship between CRT with IRE1α

Parameter	CRT		r rank	P
	Low (***n*** = 31)	High (***n*** = 50)		
IRE1α			−0.278	0.012
Negative (*n* = 43)	11	32		
Positive (*n* = 38)	20	18		

In addition, CRT high expression or IRE1α positive expression were associated with the poor and better prognosis, respectively (*P =* 0.012; *P* = 0.035, respectively) (Fig. 8g and h). Moreover, patients with high CRT and negative IRE1α expression had a much worse survival (*P* = 0.011) (Fig. 8i). Taken together, the close interaction of CRT and IRE1α coordinately participated in the aggressive clinical stages and prognosis of PC patients.

## Discussion

Due to the strong peripancreatic invasion and distant metastasis as well as insensitivity to chemotherapy, the prognosis of PC patients is extremely poor, with a 5-year survival rate of less than 5% [25]. It is now well recognized that EMT is the “booster” for the malignant progression of PC [26], which is implicated in enhancing invasion and metastasis in malignancies. EMT is typically characterized by the activation of ZEB1, N-cadherin, Vimentin, Snai1, Slug and Caveolin-1, and the downregulation of epithelial markers E-cadherin and ZO-1 expression [27, 28]. Our previous study confirmed that CRT silencing inhibited EGF-induced EMT in PC via Integrin/EGFR-ERK/MAPK signaling [4]. In present study, we first demonstrated that CRT mediated EMT via regulating intracellular free Ca^2+^ mediated acute and chronic ERS in PC, which, to our knowledge, has not been reported yet.

CRT, initially identified as a ubiquitous ER protein in 1974 [29], has diverse biological functions in cellular metabolism and biology, depending on the different locations inside and outside the ER [30]. CRT regulates Ca^2+^ homeostasis and molecular chaperoning activity within the ER [31]. However, CRT located in the cytoplasm plays contradictory roles in cancer progression [32]. For example, CRT exhibits an oncogenic role in lung [33], breast [34, 35], gastric [36], hepatic [37] and bladder cancers [38], as well as in oral [39] and esophageal squamous cell carcinoma [40, 41], but acts as a tumor suppressor in neuroblastoma [42, 43]. Meanwhile, the role of CRT remains inconclusive in colon [44–47], prostate [48, 49] and ovarian cancers [50, 51].

Ca^2+^ is mainly stored in ER lumen, which is a critical regulator involved in cancer progression [52]. Accumulating evidences indicate that transient elevation of intracellular free Ca^2+^ can promote tumor cell migration and invasion. Conversely, sustained free Ca^2+^ stimulation might lead to the cell apoptosis and death [53]. The disruption of Ca^2+^ homeostasis also triggers ERS, that is closely associated with EMT [54]. For example, EMT is induced in breast cancer cells in parallel with the increase of cytosolic Ca^2+^, whereas chelating Ca^2+^ blocked the induction of EMT markers [55]. CRT is considered as an intracellular Ca^2+^ regulator. It contains two Ca^2+^-binding domains: C-domain with a low affinity and high capacity region, and the P-domain with a high affinity and low capacity region [56]. Thus, CRT deficiency generally leads to the decrease of intracellular Ca^2+^ storage [57, 58]. However, to our knowledge, there is no direct research involving the mechanism of CRT in regulating Ca^2+^-mediated EMT in PC. TG, as an effective inhibitor of SERCAs, causes an increase of cytoplasmic free Ca^2+^ concentration and further induces acute ERS via the depletion of Ca^2+^ from ER [59]. We first found that CRT silencing inhibited TG-induced increase of intracellular free Ca^2+^ concentration. Meanwhile, TG-induced EMT in vitro by activating the key protein targets in EMT and ERK/MPK signaling (Slug, E-cad, ZO-1 and pERK), and enhancing cell mobility, which was also reversed by CRT silencing. Slug (also known as Snail2), is the most thoroughly investigated EMT regulator [60]. As a transcription factor, Slug binds to the E-cadherin promoter to repress its transcription and triggers the steps of desmosomal disruption and cell spreading, which is the key step of the EMT process [61]. ERK signaling is also essential for EMT. An ERK-dependent epigenetic remodeling of regulatory elements results in a gene expression programme essential for driving EMT [62]. TGF-β1 activates ERK signaling, which is required for TGF-β1-mediated EMT in vitro [63]. Musashi2 promotes EGF-induced EMT in PC via ZEB1-ERK/MAPK signaling [64]. Taken together, CRT silencing inhibited TG-induced acute ERS and EMT via regulating Slug and ERK signaling in vitro. Interestingly, TG also induced IRE1α expression which was negatively regulated by CRT in vitro. IRE1α silencing enhanced TG-induced cell mobility. Thus, we next focused on the relationship between CRT and UPR in chronic ERS.

Chronic ERS produces endogenous or exogenous damage to cells and triggers an UPR response. IRE1α is the most evolutionally conserved one in UPR [65]. As an ER type I transmembrane protein, the role of IRE1α in cancers is no longer simply considered as an oncogene or tumor suppressor, but a key component of cell fate switch, depending on different cancer types [66]. IRE1α mediated apoptosis in human non-small cell lung cancer (NSCLC) A549 cells induced by a Tetramethylpyrazine analogue [67]. However, IRE1α overexpression was associated with the resistant mechanism to osimertinib in NSCLC HCC827/OSIR Cells [68]. Similarly, several studies have shown that IRE1α plays a contradictory role in colon cancer cells [69–72]. We next found that CRT was co-immunoprecipitated and co-localized with IRE1α in vitro and its stable silencing caused chronic ERS by specifically activating IRE1α independent of IRE1/XBP1 axis. It is well known that IRE1/ XBP1 axis plays a key role in mediating UPR in response to ERS [73]. IRE1α/XBP1 pathway is a potential therapeutic target for Myc-driven cancers and multiple myeloma [74, 75]. However, IRE1 also exhibits XBP1-independent biochemical activities just shown in current study and previous reports [76, 77]. We next found that IRE1α silencing promoted EMT in vitro by enhancing cell mobility and activating EMT and ERK signaling, which was significantly reversed by CRT silencing. Interestingly, ERK1/2 activation is partially IRE1-dependent in mouse embryonic fibroblast cells treated with ER stress inducer [78], while IRE1 silencing attenuated ERK1/2 activation following ER stress in gastric cancer cells [79], which is inconsistent with current study. These inconsistent results might be due to the different cell types and microenvironment. Taken together, CRT silencing inhibited IRE1α silencing-induced chronic ERS and EMT via Slug and ERK signaling in PC cells, which has not been reported, to our knowledge.

Finally, CRT silencing inhibited subcutaneous tumor size and distant liver metastasis in vivo following with the increase of IRE1α expression. In human PC samples, CRT overexpression and IRE1α positive expression was positively and negatively associated with advanced clinical progression and poor survival of PC patients, respectively. Additionally, we found a negative expression of CRT and IRE1α in PC samples, which coordinately affected the patients’ survival. These findings indicate that CRT and ERS pathways cooperatively contribute to the aggressive progression of PC.

## Conclusion

**Fig. 9:**
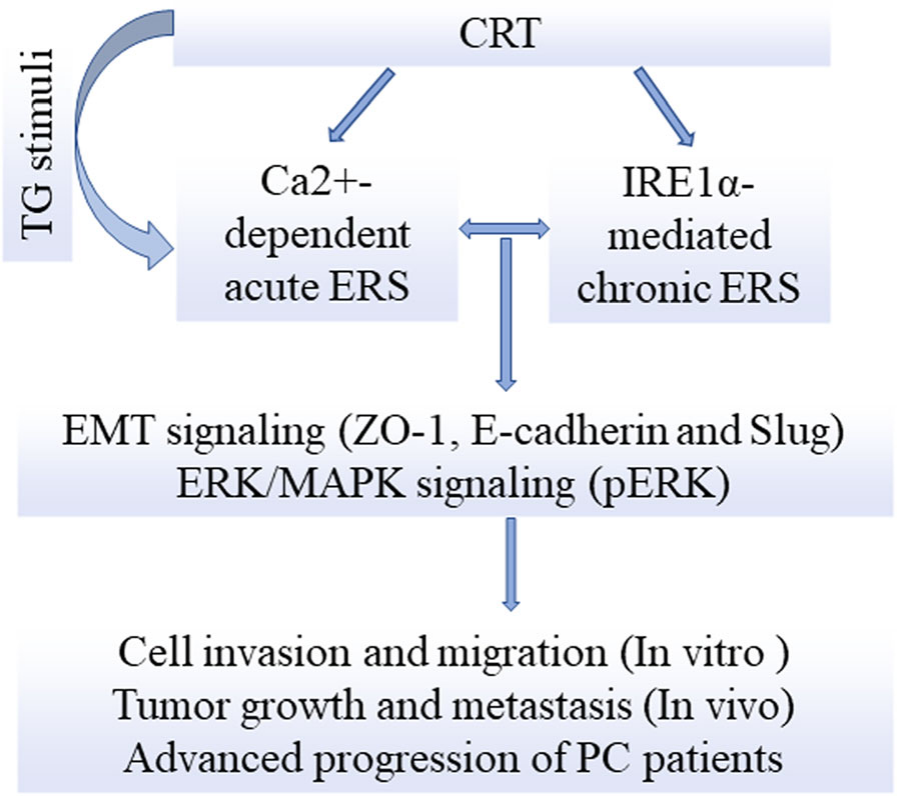
Calreticulin promotes EMT in pancreatic cancer via mediating Ca2+ dependent acute and chronic endoplasmic reticulum stress

In conclusion, for the first time, we demonstrated that CRT promoted EMT in PC via regulating TG-induced acute ERS and IRE1α-mediated chronic ERS in intracellular free Ca^2+^ dependent manner via Slug and ERK signaling (Fig. 9). However, we don’t systematically investigate the dynamic exchange between intracellular free Ca^2+^ and ER Ca^2+^ in vitro, which might be the key step to explain above multi-process property. Future studies are needed to investigate the molecular mechanisms between CRT with Ca^2+^ homeostasis and ERS mediated EMT in PC.

## Supplementary information

**Additional file 1: Supplemental Fig. 1**. The statistic data of WB in Fig. 1. **A** The quantified data of WB in Fig. 2a. **B** The quantified data of WB in Fig. 2b. **C** The quantified data of WB in Fig. 2c. **D** The quantified data of WB in Fig. 2d.

**Additional file 2: Supplemental Fig. 2**. IRE1α silencing enhanced TG-induced the increase of cell migration and invasion in vitro. **A, B** Cell invasion in Control, IRE1αsiRNA, TG and IRE1αsiRNA combing TG groups of Capan-2 (A) and SW1990 cells (B). **C, D** Cell migration in Control, IRE1αsiRNA, TG and IRE1αsiRNA combing TG groups of Capan-2 (C) and SW1990 cells (D). Data are shown as mean ± SD. *P < 0.05, **P < 0.01 versus control.

**Additional file 3: Supplemental Fig. 3**. The statistic data of WB in Fig. **5****. A** The quantified data of WB in Fig. 5a. **B** The quantified data of WB in Fig. 5b. **C** The quantified data of WB in Fig. 5c. **D** The quantified data of WB in Fig. 5d.

**Additional file 4: Supplemental Material Table 1** The target sequences of lentivirus and siRNA.

## Data Availability

Materials are available upon request.

## Abbreviations

PC: Pancreatic cancer
CRT: Calreticulin
ER: Endoplasmic reticulum
EMT: Epithelial-mesenchymal transition
ERS: Endoplasmic reticulum stress
UPR: Unfolded protein response
PERK: PKR-like endoplasmic reticulum kinase
IRE1α: Inositol-requiring enzyme 1α
ATF-6: Activating transcription factor-6
PDAC: Pancreatic ductal adenocarcinoma
ATCC: American Type Culture Collection
FBS: Fetal bovine serum
TG: Thapsigargin
SERCAs: Sarcoplasmic/endoplasmic reticulum Ca2 + −ATPases
HBSS: Hanks’ Balanced Salt Solution
IHC: Immunohistochemistry
WB: Western blot
p-PERK: Phosphorylation PKR-like endoplasmic reticulum kinase
pERK: Phosphorylation extracellular regulated protein kinases
ERK: Extracellular regulated protein kinases
XBP1: X-box-binding protein 1
CoIP: Coimmunoprecipitation
HE: Hematoxylin and eosin
NSCLC: Non-small cell lung cancer
