# Supplementary figures and images for: Calreticulin promotes EMT in pancreatic cancer via mediating Ca2+ dependent acute and chronic endoplasmic reticulum stress
Source: J Exp Clin Cancer Res. 2020 Oct 7;39:209. doi: 10.1186/s13046-020-01702-y (PMC7542892; doi:10.1186/s13046-020-01702-y)

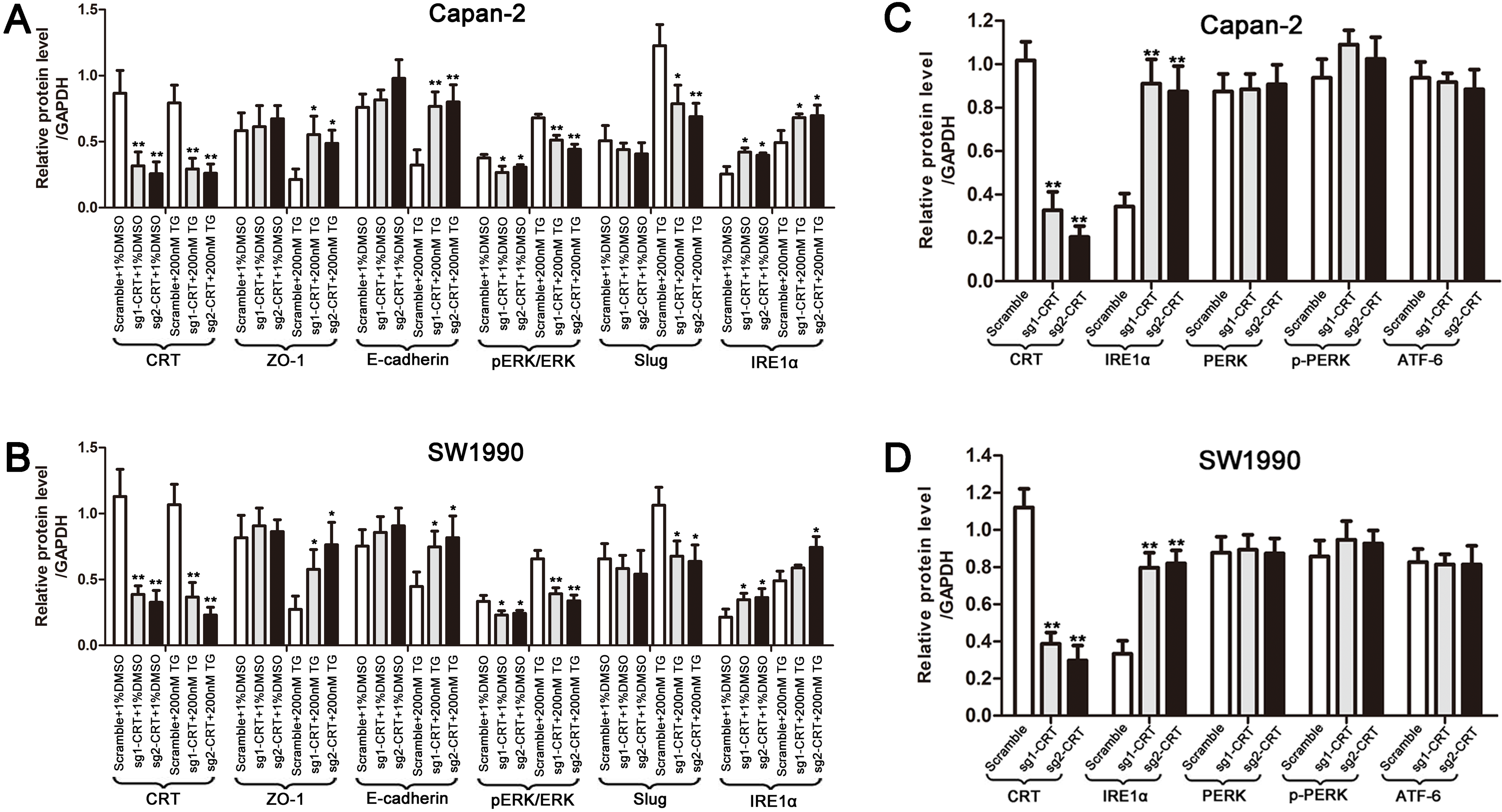

Supplement: Supplementary file 1 — Additional file 1: Supplemental Fig. 1. The statistic data of WB in Fig. 1. A The quantified data of WB in Fig. 2a. B The quantified data of WB in Fig. 2b. C The quantified data of WB in Fig. 2c. D The quantified data of WB in Fig. 2d. [file 13046_2020_1702_MOESM1_ESM.tif]

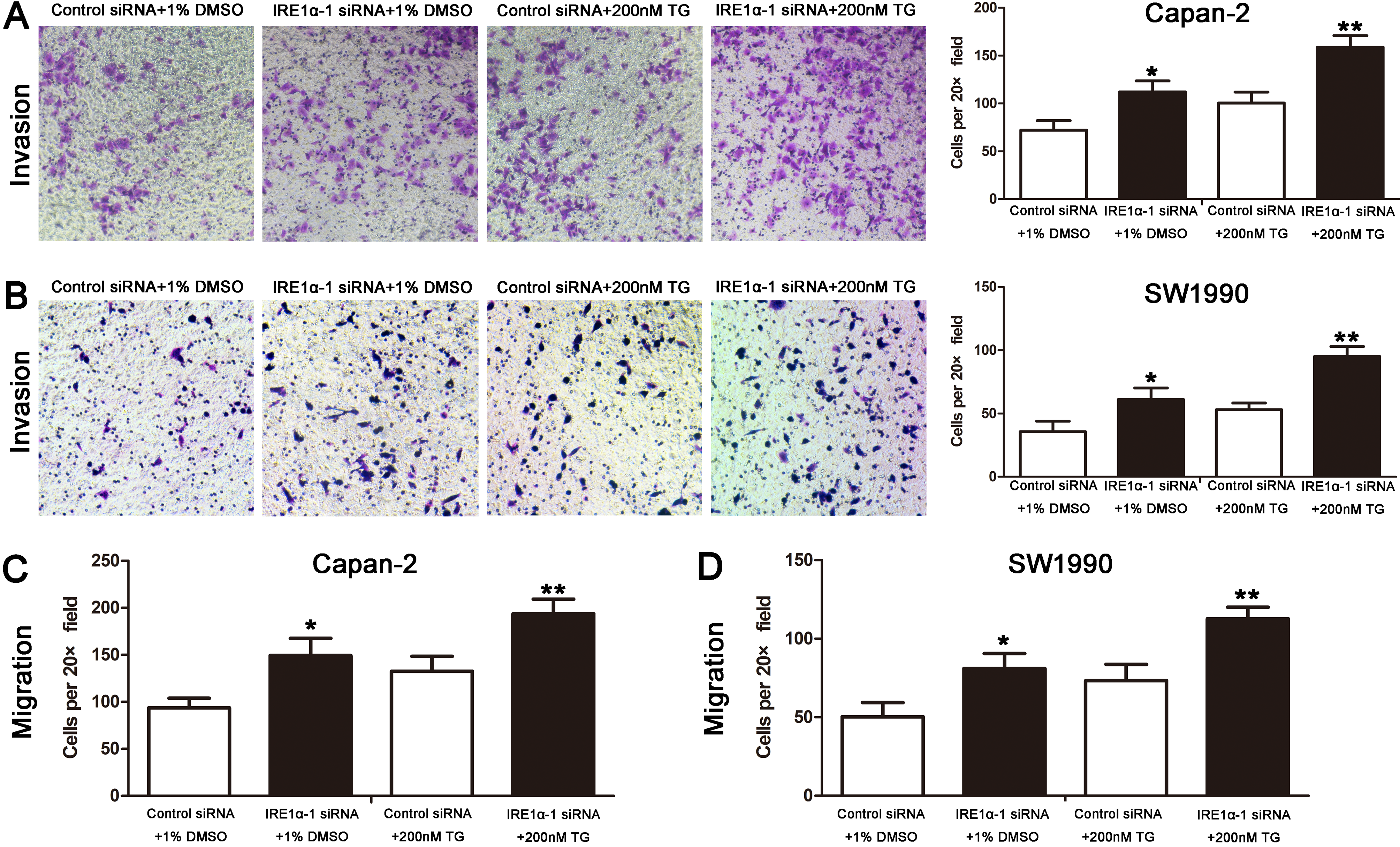

Supplement: Supplementary file 2 — Additional file 2: Supplemental Fig. 2. IRE1α silencing enhanced TG-induced the increase of cell migration and invasion in vitro. A, B Cell invasion in Control, IRE1αsiRNA, TG and IRE1αsiRNA combing TG groups of Capan-2 (A) and SW1990 cells (B). C, D Cell migration in Control, IRE1αsiRNA, TG and IRE1αsiRNA combing TG groups of Capan-2 (C) and SW1990 cells (D). Data are shown as mean ± SD. *P < 0.05, **P < 0.01 versus control. [file 13046_2020_1702_MOESM2_ESM.tif]

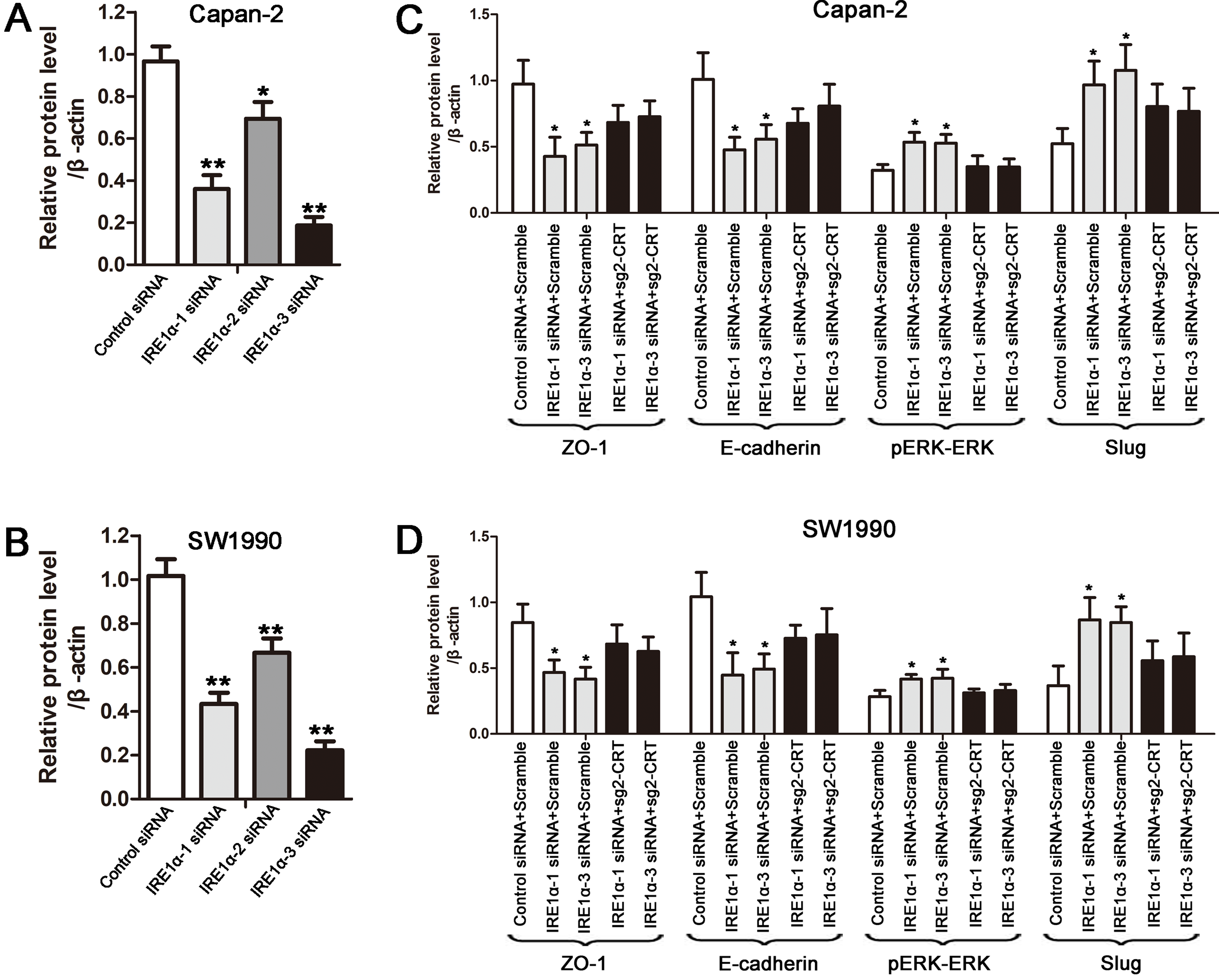

Supplement: Supplementary file 3 — Additional file 3: Supplemental Fig. 3. The statistic data of WB in Fig. 5. A The quantified data of WB in Fig. 5a. B The quantified data of WB in Fig. 5b. C The quantified data of WB in Fig. 5c. D The quantified data of WB in Fig. 5d. [file 13046_2020_1702_MOESM3_ESM.tif]
