# Supplementary material for: Calreticulin promotes EMT in pancreatic cancer via mediating Ca2+ dependent acute and chronic endoplasmic reticulum stress
Source: J Exp Clin Cancer Res. 2020 Oct 7;39:209. doi: 10.1186/s13046-020-01702-y (PMC7542892; doi:10.1186/s13046-020-01702-y)
Supplement: Supplementary file 4 — Additional file 4: Supplemental Material Table 1 The target sequences of lentivirus and siRNA. [file 13046_2020_1702_MOESM4_ESM.docx]

**Supplemental Material Table 1 The target sequences of lentivirus and siRNA.**

| **Gene** | **Oligo Name** | **Oligo Sequence** |
| --- | --- | --- |
| CRT | sg-CRT1 | －: GAAGATGACATGAACCTTCT |
|  | sg-CRT2 | +: CGAGCCTGCCGTCTACTTCA |
|  | Scramble | TTCTCCGAACGTGTCACGT |
| **Gene** | **Sense/antisense** | **Sequences** |
| IRE1α-homo-1 | Sense | CUCCGAGCCAUGAGAAAUATT |
|  | Antisense | UAUUUCUCAUGGCUCGGAGTT |
| IRE1α-homo-2 | Sense | GUCCCACUUUGUGUCCAAUTT |
|  | Antisense | AUUGGACACAAAGUGGGACTT |
| IRE1α-homo-3 | Sense | CCACACAACAUCCUCAUAUTT |
|  | Antisense | AUAUGAGGAUGUUGUGUGGTT |
| Control | Sense | UUCUCCGAACGUGUCACGUTT |
|  | Antisense | ACGUGACACGUUCGGAGAATT |
